# Wild grasses as the reservoirs of infection of rust species for winter soft wheat in the Northern Caucasus

**DOI:** 10.18699/VJ21.072

**Published:** 2021-10

**Authors:** E.I. Gultyaeva, L.A. Bespalova, I.B. Ablova, E.L. Shaydayuk, Zh.N. Khudokormova, D.R. Yakovleva, Yu.A. Titova1

**Affiliations:** All-Russian Institute of Plant Protection, Pushkin, St. Petersburg, Russia; National Center of Grain named after P.P. Lukyanenko, Krasnodar, Russia; National Center of Grain named after P.P. Lukyanenko, Krasnodar, Russia; All-Russian Institute of Plant Protection, Pushkin, St. Petersburg, Russia; National Center of Grain named after P.P. Lukyanenko, Krasnodar, Russia; All-Russian Institute of Plant Protection, Pushkin, St. Petersburg, Russia; All-Russian Institute of Plant Protection, Pushkin, St. Petersburg, Russia

**Keywords:** Puccinia triticina;., P. graminis, P. striiformis, virulence, resistance, Triticum aestivum, Lr-genes, Sr-genes, Yr-genes, Puccinia triticina, P. graminis, P. striiformis, вирулентность, устойчивость, Triticum aestivum>, Lr- гены, Sr-гены, Yr-гены

## Abstract

Common winter wheat is the main grain crop cultivated in the North Caucasus. Rust disease damage is one of the factors limiting wheat productivity. There are three species of rust in the region: leaf (Puccinia triticina), stem (P. graminis) and stripe rust (P. striiformis), and their signif icance varies from year to year. The most common is leaf rust, but in the last decade the frequency of its epiphytotic development has signif icantly decreased. At the same time, an increase in the harmfulness of stripe rust (P. striiformis) is noted. Stem rust in the region is mainly absent or observed at the end of the wheat growing season to a weak degree. Only in some years with favorable weather conditions its mass development is noted on susceptible cultivars. It is believed that the sources of infection with rust species in the North Caucasus are infested soft wheat crops, wild-growing cereals and exodemic infection carried by air currents from adjacent territories. In the North Caucasus, forage and wild grasses are affected by Puccinia species almost every year. Depending on weather conditions, the symptom expression is noted from late September to December and then from late February to May–June. Potentially, an autumn infection on grasses can serve as a source for infection of winter soft wheat cultivars sown in October. The purpose of these studies is to characterize the virulence of P. triticina, P. graminis, P. striiformis on wild cereals and to assess the specialization of causative agents to winter wheat in the North Caucasus. Infectious material represented by leaves with urediniopustules of leaf, stem and stripe rusts was collected from wild cereals (Poa spp., Bromus spp.) in the Krasnodar Territory in October–November 2019. Uredinium material from P. triticina, P. striiformis, and P. graminis was propagated and cloned. Monopustular Puccinia spp. isolates were used for virulence genetics analysis. In experiments to study the specialization of rust species from wild-growing cereals on common wheat, 12 winter cultivars were used (Grom, Tanya, Yuka, Tabor, Bezostaya 100, Yubileynaya 100, Vekha, Vassa, Alekseich, Stan, Gurt, Bagrat). These cultivars are widely cultivated in the North Caucasus region and are characterized by varying degrees of resistance to rust. Additionally, wheat material was inoculated with Krasnodar populations of P. triticina, P. striiformis, P. graminis from common wheat. In the virulence analysis of P. triticina on cereal grasses, four phenotypes (races) were identif ied: MCTKH (30 %), TCTTR (30 %), TNTTR (25 %), MHTKH (15 %), and f ive were identif ied in P. graminis (RKMTF (60 %), TKTTF, RKLTF, QKLTF, LHLPF (10 % each). Among P. striiformis isolates, three phenotypes were identif ied using the International and European sets of differentiating cultivars – 111E231 (88 %), 111E247 (6 %) and 78E199 (6 %). Using isogenic Avocet lines, 3 races were also identif ied, which differed among themselves in virulence to the Yr1, Yr11, Yr18 genes (with the prevalence of virulent ones (94 %)). Composite urediniums’ samples (a mixture of all identif ied races) of grass rust of each species were used to inoculate winter wheat cultivars. The most common winter wheat cultivars (75 %) were characterized by a resistant response when infected with P. graminis populations from common wheat and cereal grasses. All these cultivars were developed using donors of the rye translocation 1BL.1RS, in which the Lr26, Sr31, and Yr9 genes are localized. The number of winter wheat cultivars resistant to leaf rust in the seedling phase was lower (58 %). At the same time, all the studied cultivars in the seedling phase were susceptible to P. striiformis to varying degrees. The virulence analysis of the leaf, stem and stripe rust populations did not reveal signif icant differences in the virulence of the pathogens between wild-growing cereals and soft wheat. Urediniomaterial of all studied rust species successfully infested soft wheat cultivars. The results obtained indicate that grasses are rust infection reservoirs for common wheat crops in the North Caucasus.

## Abstract (ru)

Озимая мягкая пшеница – основная зерновая культура, возделываемая на Северном Кавказе. Поражение ржавчинными болезнями – один из факторов, лимитирующих урожайность пшеницы. В регионе отмечаются три вида ржавчины: бурая (Puccinia triticina), стеблевая (P. graminis) и желтая (P. striiformis), значи- мость которых варьирует по годам. Наиболее распространена бурая ржавчина, но в последнее десятилетие частота ее эпифитотийного развития существенно снизилась. При этом возрастает вредоносность желтой ржавчины (P. striiformis). Стеблевая ржавчина в регионе преимущественно отсутствует или наблюдается в слабой степени в конце вегетации пшеницы. В отдельные годы с благоприятными погодными условиями наблюдается ее массовое развитие на восприимчивых сортах. Считается, что источниками инфекции видов ржавчины на Северном Кавказе служат зараженные посевы мягкой пшеницы, дикорастущие злаки и экзо- демичная инфекция, заносимая воздушными потоками с сопредельных территорий. На Северном Кавказе практически ежегодно встречается поражение кормовых и дикорастущих злаковых трав видами Puccinia. В зависимости от погодных условий проявление симптомов отмечается с конца сентября до декабря и затем с конца февраля до мая-июня. Потенциально осенняя инфекция на травах может служить источником для заражения сортов озимой мягкой пшеницы, высеваемых в октябре. Цель настоящих исследований – охарак- теризовать вирулентность P. triticina, P. graminis, P. striiformis на диких злаках и оценить их специализацию к озимой мягкой пшенице на Северном Кавказе. Инфекционный материал, представленный листьями с уре- диниопустулами бурой, стеблевой и желтой ржавчины, был собран в Краснодарском крае на дикорастущих злаках (Poa spp., Bromus spp.) в октябре-ноябре 2019 г. В лабораторных условиях урединиоматериал P. triticina, P. striiformis и P. graminis был размножен и клонирован. Монопустульные изоляты видов Puccinia использова- ли для анализа вирулентности. В экспериментах по изучению специализации видов ржавчины с дикорасту- щих злаков на мягкой пшенице задействовали 12 озимых сортов: Гром, Таня, Юка, Табор, Безостая 100, Юби- лейная 100, Веха, Васса, Алексеич, Стан, Гурт, Баграт. Эти сорта широко возделываются в Северо-Кавказском регионе и характеризуются разной степенью устойчивости к ржавчинам. Дополнительно материал пшеницы инокулировали краснодарскими популяциями P. triticina, P. striiformis, P. graminis с мягкой пшеницы. В анализе вирулентности P. triticina на злаковых травах выявили четыре фенотипа (расы): MCTKН (30 %), ТCTTR (30 %), ТНТTR (25 %), MHTKН (15 %), а P. graminis – пять фенотипов: RКMTF (60 %), TКТTF, RКLTF, QKLTF, LHLРF (по 10 %). Среди изолятов P. striiformis с применением международного и европейского наборов сортов-дифференциа- торов определены три фенотипа: 111Е231 (88 %), 111Е247 (6 %) и 78Е199 (6 %). С использованием изогенных линий Avocet также идентифицированы три расы, которые различались между собой по вирулентности к генам Yr1, Yr11, Yr18 (с доминированием вирулентных (94 %)). Сборные урединиообразцы (смесь всех иден- тифицированных рас) каждого вида ржавчины со злаковых трав были задействованы для инокуляции сортов озимой пшеницы. Большинство сортов озимой мягкой пшеницы (75 %) характеризовались устойчивой реакцией при заражении популяциями P. graminis с мягкой пшеницы и злаковых трав. Все эти сорта созданы с участием доноров ржаной транслокации 1BL.1RS, в которой локализованы гены Lr26, Sr31 и Yr9. Число сортов озимой пшеницы, устойчивых в фазе проростков к бурой ржавчине, было ниже (58 %). При этом все изученные сорта в фазе проростков в разной степени были восприимчивы к Р. striiformis. Проведенный анализ вирулентности популяций возбудителей бурой, стеблевой и желтой ржавчины не выявил существенных раз- личий в вирулентности патогена на дикорастущих злаковых травах и мягкой пшенице. Урединиоматериал всех изученных видов ржавчины успешно заражал сорта мягкой пшеницы. Полученные результаты указыва- ют на то, что злаковые травы являются резерваторами инфекции ржавчин для посевов мягкой пшеницы на Северном Кавказе.

## Introduction

Common winter wheat is the main grain crop cultivated in the North Caucasus. Its sown area in this region is more than 7 million hectares (ha), including 1.5 million ha in the Krasnodar Territory, 3 million ha – in the Rostov region, 2.5 million ha ones in the Stavropol Territory and other republics. Leaf disease is one of the factors limiting wheat yield.

At the same time, an increase in the importance of stripe rust (P. striiformis West.) is noted in the region, which is associated with climate change (long warm autumn, mild winters, lack of soil freezing, prolonged cool springs) (Ablova et al., 2012). Stripe rust is more harmful than leaf rust and can reduce the yield by up to 30 % (Sanin, 2012).

Stem rust (P. graminis Pers. f. sp. tritici Erikss. & E.) is mainly absent in the region or observed at the end of the wheat growing season to a weak degree. Only in some years with favorable weather conditions its mass development was noted on susceptible cultivars. This is due to wide cultivation in the region of winter wheat cultivars with the Sr31 gene, which to this day remains highly effective in protecting against stem rust in Russia. In addition, in the breeding process, the growing season duration of modern cultivars is significantly reduced, which contributes to their escape from the disease (Ablova et al., 2012).

It is believed that the sources of rust species infection in the North Caucasus are the infected crops of soft wheat, wild cereals and exodemic infection carried by air currents from adjacent territories. Winter wheat is sown in September- October. Harvesting takes place from the second half of June to the end of July. Accordingly, rust pathogens uredinioinfection on winter wheat can persist from October to June. Forage and wild-growing grasses (Bromus, Poa, Festuca, Agropyron, Elimus, Aegilops, Hordeum, Agrostis spp.) can serve as additional infection reservators. Transboundary transfer of rust pathogens urediniospores to the territory of the North Caucasus is possible from Turkey, Iraq, and Iran (Sanin, 2012). According to L.K. Anpilogova et al. (1995), in the North Caucasus, in the epiphytotic years, the infection of wheat stripe rust pathogen appears due to its migration from the Transcaucasia territory to Dagestan, Ossetia, Ingushetia, Kabardino-Balkaria, the foothills and the adjacent steppe of Stavropol and Krasnodar regions.

It is believed that the sources of rust species infection in the North Caucasus are the infected crops of soft wheat, wild cereals and exodemic infection carried by air currents from adjacent territories. Winter wheat is sown in September- October. Harvesting takes place from the second half of June to the end of July. Accordingly, rust pathogens uredinioinfection on winter wheat can persist from October to June. Forage and wild-growing grasses (Bromus, Poa, Festuca, Agropyron, Elimus, Aegilops, Hordeum, Agrostis spp.) can serve as additional infection reservators. Transboundary transfer of rust pathogens urediniospores to the territory of the North Caucasus is possible from Turkey, Iraq, and Iran (Sanin, 2012). According to L.K. Anpilogova et al. (1995), in the North Caucasus, in the epiphytotic years, the infection of wheat stripe rust pathogen appears due to its migration from the Transcaucasia territory to Dagestan, Ossetia, Ingushetia, Kabardino-Balkaria, the foothills and the adjacent steppe of Stavropol and Krasnodar regions.

In the North Caucasus, forage and wild-growing cereal grasses are annually affected by Puccinia species (see the Figure). Depending on weather conditions, the symptom expression is observed from late September up to December and then from late February up to May-June. Potentially, an autumn infection on grasses can serve as a source for infection of common winter wheat cultivars sown in October.

The purpose of these studies is to characterize the virulence of P. triticina, P. graminis, P. striiformis on wild cereals and to assess the specialization of causative agents to winter wheat in the North Caucasus.

## Materials and methods

Infectious material, represented by leaves with urediniopustules of leaf, stem and stripe rust, was collected on wild cereal grasses (Poa spp., Bromus spp.) in the Krasnodar Territory in October-November 2019 (see the Figure). The analysis used 18 uredinium samples. The infectious material was dominated by P. triticina and. P. striiformis. P. graminis pustules were of limited abundance. A total of 20 monopustular isolates of P. triticina, 16 of P. striiformis, and 10 of P. graminis were studied.

**Fig.**
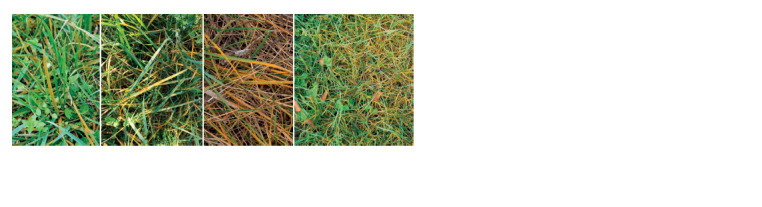
Wild cereal grasses (Poa spp., Bromus spp.) with rust species infection (Krasnodar Territory, November 2019).

Infectious material reproducing and obtaining monopustular isolates of Puccinia sp. The universally susceptible Michigan Amber winter wheat cultivar was used to propagate an inoculum of rust species and obtain monopustular isolates. Under laboratory conditions, the uredinium material of P. triticina and P. graminis was propagated and cloned using the method of leaf segments placed in a benzimidazole solution (0.004 %) (Mikhailova et al., 1998). The urediniospores of each monopustular isolate were microscopically investigated to confirm the Puccinia species and prevent contamination.

Since the viability of P. striiformis urediniospores in the herbarium material is short, the populations were “reanimated” on leaf segments (Mikhailova et al., 1998). For this purpose, the leaves of herbs with urediniopustules were cut into pieces of 5–8 cm and placed in Petri dishes, on the bottom of which two slides were placed. The ends of the leaf segments were covered with cotton wool soaked in a solution of benzimidazole (0.004 %), the Petri dishes were closed and placed in a refrigerator (temperature 3–5° C) for 2–4 days. Such a technique made it possible to stimulate theresumption of pathogen sporulation. Subsequent propagation of the pathogen was carried out on 10–12-day old wheat plants grown in vessels with soil using the microchamber method. For this purpose, the pieces of leaves with urediniopustules were applied to the leaves and fixed with cling film. Vessels with plants were sprayed with water, covered with plastic wrap frames, and placed into a dark chamber at 10° C for 18–20 h. Then, frames and microchambers with infectious material were removed. Plants were transferred to a Versatile Environmental Test Chamber MLR-352H (SANYO Electric Co., Ltd.), where they were incubated in the light (10,000–20,000 lx) at 16° С for 16 h and then in the dark at 10° С for 8 h (70 % humidity). The symptoms expression was observed 12–18 days after infesting.

Merck vacuum pump (Millipore) (220 V/50 Hz) with special nozzles (1 clone/1 nozzle) was used to collect spores of monopustular rust isolates.

Virulence analysis. The virulence analysis of the populations was carried out using 10–12-day old plants from differentiating lines grown in vessels with soil (1 set/1 monopustular isolate). Plants were sprayed with a spore suspension in a specialized liquid NOVEC 7100, covered with a polyethylene frame to produce a humid chamber and kept in the dark at 20–23° С for leaf and stem rust, at 10° С for stripe rust. After 12 h, the polyethylene was removed and special perforated insulators were pulled over the frames to prevent contamination. Sets of differentiating lines infected by P. triticina and P. graminis were incubated in a light installation at 20–23° С (photoperiod was 16 h/ daytime (illumination 10,000–15,000 lx)/8 h/nighttime), those infected by P. striiformis – in the climatic chamber according to the parameters described above.

To study the virulence of the leaf rust causative agent isolates, were used the Thatcher (Tc) lines with genes Lr1, Lr2a, Lr2c, Lr3a, Lr3bg, Lr3ka, Lr9, Lr11, Lr14a, Lr14b, Lr15, Lr16, Lr17a, Lr18, Lr19, Lr20, Lr24, Lr26 and Lr30; to study the virulence of the stem rust causative agent isolates – the Marquis (Mq) lines with genes Sr5, Sr6, Sr7b, Sr8a, Sr9a, Sr9b, Sr9g, Sr9e, Sr9d, Sr10, Sr11, Sr17, Sr21, Sr24, Sr30, Sr31, Sr36, Sr38, SrTmp and SrMcN.

The analysis of the stripe rust pathogen was carried out using International (Chinese 166 (Yr1), Lee (Yr7, Yr+), Heines Kolben (Yr2+Yr6 ), Vilmorin 23 (Yr3), Moro (Yr10, YrMor), Strubes Dickkopf (YrSD, Yr+), Suwon 92/Omar (YrSu, Yr+) and European (Hybrid 46 (Yr4, Yr+), Reichersberg 42 (Yr7, Yr+), Heines Peko (Yr6, Yr+), Nord Desprez (Yr3, YrND, Yr+), Compair (Yr8, Yr19), Carstens V (Yr32, Yr+), Spaldings Prolific (YrSP, Yr+), Heines VII (Yr2, Yr+)) sets of differentiating cultivars, as well as the Avocet (Aс) lines with genes Yr1, Yr5, Yr6, Yr7, Yr8, Yr9, Yr10, Yr11, Yr12, Yr15, Yr17, Yr18, Yr24, Yr26, YrSk(27), YrAR, YrSp. Seed material of cultivars and differentiating lines was kindly provided by A.S. Rsaliev (Research Institute of Biological Security Problems, Kazakhstan).

To designate the races of leaf and stem rust, the North American letter abbreviation was used, according to which the lines were combined into groups (4 lines each) (Long, Kolmer, 1989). The set of lines for stem rust included the lines with genes: Sr5, Sr21, Sr9e, Sr7b (group 1); Sr11, S6, Sr8a, Sr9g (group 2); Sr36, Sr9b, Sr30, Sr17 (group 3); Sr9a, Sr9d, Sr10, SrTmp (group 4); Sr24, Sr31, Sr38, SrMcN (group 5) (Skolotneva et al., 2020); for leaf rust – Lr1, Lr2a, Lr2c, Lr3 (group 1); Lr9, Lr16, Lr24, Lr26 (group 2); Lr3ka, Lr11, Lr17, Lr30 (group 3); Lr2b, Lr3bg, Lr14a, Lr14b (group 4); Lr15, Lr18, Lr19, Lr20 (group 5) (Gultyaeva et al., 2020).

Determination of the stripe rust pathogen races was carried out using International and European differential sets. Decimal nomenclature was used for designation. It is based on a binary designation system for infection types (resistant type of reaction (R) is designated as 0, susceptible (S) as 1) and a decimal designation system for each cultivar (the first differentiating cultivar is 20, the second one is 21, the third one is 22, etc.). Due to the fact that two sets of differentiating cultivars, International and European, were used, when naming a race, first the number according to the International set was written, then the number according to the European one with the prefix E (for example, 1E3) (Gultyaeva, Shaydayuk, 2020).

Immunological studies of winter wheat cultivars in laboratory and field conditions. Krasnodar P. triticina, P. graminis and P. striiformis populations originating from cereal grasses and soft wheat were used for inoculation of the twelve cultivars of common winter wheat: Grom, Tanya, Yuka, Tabor, Bezostaya 100, Yubileynaya 100, Vekha, Vassa, Alekseich, Stan, Gurt, Bagrat. These cultivars are widely cultivated in the North Caucasus region and are characterized by varying degrees of resistance to rust.

Under laboratory conditions, the plants were grown in plastic containers (5–8 grains of each cultivar). At the first leaf phase (10–12-day plants), they were sprayed with each rust species spore suspension in NOVEC 7100. The incubation of infected plants was carried out according to the parameters described above.

The reaction type of differentiating lines and wheat cultivars on leaf rust infection was assessed at 8–10 days according to the E.B. Mains and H.S. Jackson scale (1926), on stem rust infection – at 10–12 days according to the scale of E.C. Stakman et al. (1962), on stripe rust infection – at 16–18 days on the scale of G. Gassner and W. Straib (1928). Plants with scores 0, 1, 2 were classified as resistant, with scores 3, 4, X – as susceptible.

To produce artificial infectious backgrounds in the field conditions of the National Grain Center (Krasnodar Territory), the following methods were used: the plants were inoculated by spraying with an aqueous spore suspension with Tween 80 adhesive, and a wet chamber was made using plastic bags (for the leaf and stripe rusts). Plants were infected with stem rust using a syringe. The consumption rate or infectious load was 10 mg/m2 of spores for the leaf rust pathogens, 20 mg/m2 for the stem and stripe rusts. For successful infestation of leaf rust, the temperature should be at least 15° C, infestation of stem rust – 18° C, infestation of stripe rust – 10° C. Plants were infected with stripe rust in the booting phase, with leaf and stem rust – in the booting and then in the earing phase.

Resistance to rust species was determined by qualitative (reaction type) and quantitative indicators (damage intensity). The reaction type to leaf rust was determined on the E.B. Mains and H.S. Jackson scale (1926), to yellow (stripe) rust – on the G. Gassner and W. Straib scale (1928), to stem rust on the scale of E.C. Stakman et al. (1962). The plants’ damage was visually determined: leaf rust infection on flag and pre-flag leaves, stripe rust infection on three upper leaves, stem rust infection on two upper internodes, sheaths of flag and pre-flag leaves. The damage intensity by leaf and stem rusts was determined according to the Peterson scale, and by stripe rust – the modified Cobb scale (McIntosh et al., 1995). Damage intensity registration by rust types was carried out in the period from earing up to milky-wax ripeness.

## Results and discussion

The urediniospores of P. triticina, P. graminis, and P. striiformis from wild cereals successfully infected the universally susceptible winter wheat cultivar Michigan Amber, which made it possible to carry out population genetic studies of pathogens and immunological studies of wheat cultivars. In the analysis of P. triticina virulence, 20 monopustular isolates were studied and four races (phenotypes) were identified: MCTKH (30 %), TCTTR (30 %), TNTTR (25 %), MHTKH (15 %). All isolates were avirulent to the Thatcher lines with genes Lr9, Lr19, Lr24 and virulent to Lr1, Lr3a, Lr3bg, Lr3ka, Lr11, Lr14a, Lr14b, Lr17a, Lr18, Lr20, Lr26, Lr30. Frequencies variation was observed on the lines TcLr2a, TcLr2b, TcLr2c, TcLr15 (55 % virulence) and TcLr16 (40 %).

When analyzing the P. graminis population, a higher phenotypic diversity was observed. Five races (RКMTF (60 %), TKTTF, RКLTF, QKLTF, LHLРF (10 % each)) were identified among the 10 monopustular isolates studied. All isolates were avirulent to the Marquis lines with genes Sr9e, Sr11, Sr24, Sr30, Sr31 and virulent to genes Sr5, Sr6, Sr9a, Sr9g, Sr10, Sr36, Sr38, SrTmp, SrMcN. Variability in reaction types was observed on the lines Sr7b, Sr8a, Sr9b, Sr9e, Sr9d, Sr17, Sr21.

Virulence to stripe rust was studied using 16 monopustular isolates. All isolates were avirulent to the Moro, Nord Desprez, Compair differentiating cultivars and the Avocet lines with genes Yr5, Yr8, Yr10, Yr12, Yr15, Yr17, Yr24, Yr26 and virulent to the cultivars Lee, Heines Kolben, Vilmorin 23, Hybrid 46, Reichersberg 42, Suwon 92/Omar, Heines Peko, Spaldings Prolific, Heines VII as well as to the lines with genes Yr6, Yr10, YrSk(27), YrAR, YrSp. Virulence variations were noted for cultivars Chinese 166, Strubes Dickkopf, Carstens V and the lines AcYr1, AcYr11 and AcYr18. According to the International and European sets of differentiating cultivars, P. srtriiformis isolates were represented by the races 111E231 (88 %), 111E247 (6 %), and 78E199 (6 %). In the virulence analysis using the Avocet lines, three phenotypes were identified, differing from each other in virulence to Yr1, Yr11, Yr18 (with the prevalence of virulent ones (94 %)).

Combined populations of each rust species from cereal grasses were used to infect winter wheat cultivars widely cultivated in the region (Table 1). The inoculum included isolates of all races of the pathogen identified in the virulence assay. Additionally, the studied cultivars were infected with Krasnodar populations of P. triticina, P. striiformis, P. graminis from soft wheat. The P. triticina population was avirulent to the Thatcher lines with Lr genes: 9, 16, 19, 24 and virulent to Lr genes: 1, 2a, 2b, 2c, 3a, 3bg, 3ka, 10, 14a, 14b, 15, 17, 18, 20, 26, 30; P. striiformis population was avirulent to Yr genes: 5, 10, 15, 17, 24, 26 and virulent to Yr genes: 1, 3, 4, 6, 7, 8, 9, 18, 32, Sp; P. graminis population was avirulent to Sr genes: 24, 30, 31, and virulent to Sr genes: 5, 6, 7b, 8a, 9a, 9e, 9d, 10, 21, 36, 38, McN, Tmp.

**Table 1.**
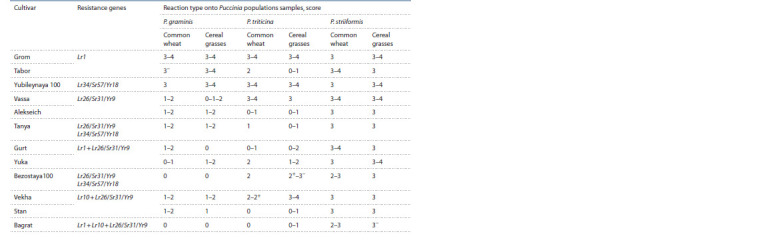
Resistance of common wheat cultivars to rusts at the seedling stage

Most cultivars of winter soft wheat (75 %) were characterized by a resistant reaction when infected with P. graminis populations from soft wheat and cereal grasses (see Table 1). Many of them have the 1BL.1RS rye translocation, in which the Lr26, Sr31, and Yr9 genes are localized. Despite the fact that the efficiency of the Sr31 gene has been overcome in a number of countries in the world, the virulence to this gene in the North Caucasus region has not yet been identified. At the same time, the rapid range expansion of the races of Ug99 group, virulent to Sr31, and their detection in territories close to the North Caucasus of Russia (for example, Iran) presupposes continuous monitoring of this pathogen populations and improving genetic protection (Nazari et al., 2009).

The number of winter wheat cultivars resistant to leaf rust in the seedling phase was lower (58 %). These included cultivars Tabor, Alekseich, Tanya, Gurt, Yuka, Stan, Bagrat. Cultivars Bezostaya 100 and Vekha were moderately resistant (score 2–2+) to the population of the pathogen from common wheat, but susceptible to the population from cereal grasses. The Lr26 gene of these cultivars has long lost its effectiveness in Russia. At the same time, pyramiding of this gene with other genes is effective (Sibikeev et al., 2011). The cultivars Grom, Yubileynaya 100 and Vassa belonged to the group susceptible to leaf rust.

All studied cultivars showed various degrees of susceptibility to P. striiformis populations from cereal grasses and common wheat (scores 2–3, 3, 3–4). This indicates that genes Yr9 and Yr18, which are widely represented in winter wheat cultivars handled in the North Caucasus, are not effective in protecting against stripe rust in the seedling and tillering phases. Accordingly, such cultivars can accumulate aerogenic infection from cereal grasses and under favorable weather conditions contribute to its appearrance and mass development.

These researches revealed a high diversity in the studied isolates of Puccinia species. The races ratio analysis of the rust species on cereal grasses and on wheat crops was of considerable interest. The P. triticina races identified on cereal grasses in these researches (MCTKH, MHTKH, TCTTR, THTTR) are regularly noted in analyses of North Caucasian and other Russian populations (Kolmer et al., 1915; Gultyaeva et al., 2020). In 2019, the P. srtriiformis 111E247 race dominated Krasnodar and Leningrad pathogen populations on soft wheat, and the 78E199 race dominated the Novosibirsk population (Gultyaeva, Shaidayuk, 2020). The P. graminis races RKMTF, TKTTF, RKLTF, QKLTF, LHLРF are similar in virulence to those identified on common wheat in samples of North Caucasian and European populations of this pathogen (Skolotneva et al., 2013; Sinyak et al., 2014).

Our obtained results are consistent with the data presented in the literature (Budarina, 1955; Borisenko, 1970; Lesovoy, Tereshchenko, 1972; Krayeva, Matviyenko, 1974; Paichadze, Yaremenko, 1974; Berlyand-Kozhevnikov et al., 1978; Popov, 1979; Hovmøller et al., 2011; Cheng et al., 2016). In the 1969–1972 research of P. striiformis uredinium samples collected on a wide set of wild cereals in the North Caucasus, the 20, 31, 19, 9, etc. races were identified, which are also highly specialized to common wheat (Krayeva, Matviyenko, 1974). The analysis of stripe rust inoculum collected from five cereal grass species (creeping wheatgrass, fibrous regneria, siberian wild rye, cock’s-foot grass, redtop) in 1973 in the Altai Territory revealed that isolates from siberian wild rye, fibrous regneria and creeping wheatgrass perform virulence to soft wheat (Popov, 1979). Among the specimens of P. striiformis collected on wild cereals in 1968–1972 in the vast territory of Georgia eight revealed races 20, 31, 40, 19, 42A2, 25, 13, 20A2 are also specialized for soft wheat (Paichadze, Yaremenko, 1974).

A high genetic diversity of stripe rust isolates in terms of virulence on wild-growing cereals has also been shown in other countries (Hovmøller et al., 2011; Cheng et al., 2016). This is due to the balanced genetic diversity of hosts and their parasites in natural biocenoses. For example, among the isolates of P. striiformis obtained from 11 species of wild-growing cereals in the USA, isolates that are virulent to common wheat (f. sp. tritici), barley (f. sp. hordei), both of these species, rye, triticale and other cereals were identified.

Similar results were obtained when studying the causative agent of stem rust in 2000–2009 in the Central region of Russia (Skolotneva et al., 2013). A high similarity in the composition of the pathogen populations on soft wheat and wild cereals was determined. Analysis of the long-term dynamics of the main P. graminis races showed that the phenotypes that dominate on common wheat and other cultivated cereals in some years do not completely disappear in unfavorable seasons, but remain on wild cereals (Skolotneva et al., 2013).

According to V.M. Berlyand-Kozhevnikov et al. (1978), the main (maternal) population of the leaf rust pathogen in southern Dagestan is a set of pathogen clones that parasitize on wheatgrass and other perennial wild cereals throughout the year. In early spring, and sometimes in autumn, the disease also appears on various annual cereals. The spread of the pathogen population from perennial cereals to wheat crops begins with the development of clones that can parasitize on the corresponding host plants. This hypothesis was confirmed by studying the specialization of the leaf rust pathogen in Ukraine forest-steppe conditions in the 1970s (Lesovoy, Tereshchenko, 1972). The isolates of the fungus collected from cereal grasses successfully infested common wheat cultivars and were represented by five races, among which the race 77 dominated. Its frequency in rust samples from Elitrigia repens, Bromus tectorum, Festuca pratensis, Poa angutifolia reached 100 %. Other races were found singularly: the race 6 was revealed in P. trivialis samples, the race 4 was revealed in A. imbricatum, the races 130 and 144 were revealed in B. mollis. The fungus population on common wheat had these races in unsignificant numbers. N.A. Budarina (1955) showed that Ae. cylindrica, cheat grass (B. tectotium) and narrow-ear wheatgrass (Ag. cristatum var. imbricatum) can serve as leaf rust reservators in the Crimea. Leaf rust infection obtained from these species successfully infected wheat. A.N. Borisenko (1970), when studying P. triticina populations on wild cereals in Kazakhstan, Kyrgyzstan, and Western Siberia, identified 10 races on them, and all these races were recorded on common wheat.

Most species of wild-growing cereals are perennials, so rust pathogens can persist in the form of urediniopustules or urediniomycelium for a long period. During the 2019–2020 growing season in the Krasnodar Territory, an air temperature excess over the long-term average values was noted in every month, except April and May. The winter wheat growing season practically did not stop both in autumn and winter.

Despite the fact that in the fall of 2019 in the central and southern foothill zone of the Krasnodar Territory, the foothill zone of the Stavropol Territory, powerful infectious potential of rust pathogens with a dominance of stripe rust was formed on wild and weed cereals, in autumn and winter surveys of breeding and industrial crops of winter wheat no stripe rust symptoms were detected. This is due to insufficiently favorable weather conditions for the wheat infection. Due to the acute limit of moisture supply in spring, low relative humidity, as well as windy weather, the spread and development of phytopathogens on cereal grasses stopped. At the same time, in the 2020 fall, like in 2019, the appearance of rust on wild and forage cereal grasses was noted again, but much less developed than in 2019. This confirms the stability in the preservation of uredinioinfection of rust species in natural biocenoses as well as the potential contamination of grain crops under favorable weather conditions.

Winter wheat cultivars used in these studies to assess the specialization of rust pathogens are leading in the North Caucasus in terms of sown area. As an example, Tanya cultivar occupies 18 % of the total area under winter wheat in the Krasnodar Territory and has been cultivated in production for more than 15 years; Grom occupies 15 %, Alekseich – 9.5 %, Yuka – 9 % of the winter wheat total sowing area. These cultivars are characterized by different levels of rust resistance when artificially infested in the field. The cultivars Alekseich and Bezostaya 100 have group resistance to three rust species. They have been approved for handling in production since 2017. The Tanya is characterized by high field resistance to Puccinia spp. The cultivars Bagrat, Vekha, Vassa, Stan, Tabor, Yubileynaya 100 are classified as moderately resistant to leaf rust. The intensity of their damage varies from 10 up to 40 % with moderately resistant and moderately susceptible reaction types. The Grom is a rust susceptible cultivar. The cultivars with Sr31 in their haplotype are resistant to stem rust: Vassa, Vekha, Bagrat, Stan – the degree of their damage does not exceed 10 % with resistant and moderately resistant reaction types. In cultivars Gurt and Yuka with similar genetic material, the damage intensity is 30 and 40 %, respectively, with a moderately susceptible reaction type. The Tabor and Yubileynaya 100 show susceptibility to stem rust. The Vekha and Bagrat cultivars have moderate resistance to stripe rust. The average rust incidence on these cultivars at the infectious site of the National Center of Grain during artificial 2018–2020 infection is presented in Table 2.

**Table 2.**
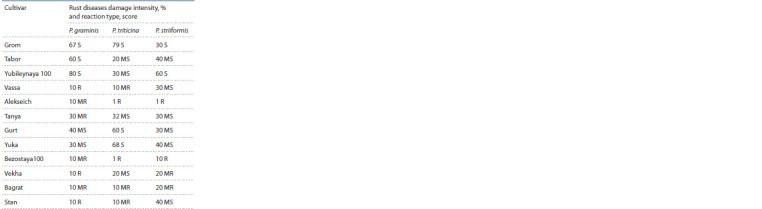
Immunological characteristic of common wheat cultivars in Krasnodar region under artif icial infection conditions (2018–2020) Notе. Reaction type: R – 1 score, MR – 2 scores, MS – heterogeneous reaction type X (2–3), S – 3–4 scores.

There were no significant differences in the infection of the studied cultivars by leaf and stripe rust against an artificial infectious background in 2018–2020 compared with the previously presented characteristic (Bespalova et al., 2020). For most cultivars, the results of field assessments correlated with those obtained in the seedling phase.

Immunological studies of winter common wheat cultivars show that despite comparatively similar haplotypes for resistance genes to rust species, the immune activity and its duration are diverse even with the “antimonopoly” law, which effectively works in the North Caucasus agrophytocenoses. This is due to the growing season duration, the period of “attacking”, as well as the infectious load during the critical phases of the host plant ontogenesis for infesting. For stable protection of wheat plants against Puccinia spp. it is necessary to use genes that determine various mechanisms of resistance.

## Conclusion

The analysis of the racial composition and virulence of the pathogens’ populations of leaf, stem and stripe rust indicates that in the conditions of Russian North Caucasus wild cereals are reservators of rust species and, under favorable weather conditions, can serve as a source of infection for common wheat crops and other cultivated cereals. The high diversity of the racial composition of the pathogen in natural cenoses and the wide specialization of Puccinia isolates imply the continuous evolution of the pathogen due to the emergence of new mutations in virulence and somatic hybridization, which should be considered in breeding for cereals resistance to rust.

## Conflict of interest

The authors declare no conflict of interest.
